# Multi-Exposure Image Fusion Algorithm Based on Improved Weight Function

**DOI:** 10.3389/fnbot.2022.846580

**Published:** 2022-03-08

**Authors:** Ke Xu, Qin Wang, Huangqing Xiao, Kelin Liu

**Affiliations:** College of Computer Science and Technology, Chongqing University of Posts and Telecommunications, Chongqing, China

**Keywords:** high dynamic range image, multi-scale decomposition, multi-exposure images, image fusion, Laplacian pyramid (LP)

## Abstract

High-dynamic-range (HDR) image has a wide range of applications, but its access is limited. Multi-exposure image fusion techniques have been widely concerned because they can obtain images similar to HDR images. In order to solve the detail loss of multi-exposure image fusion (MEF) in image reconstruction process, exposure moderate evaluation and relative brightness are used as joint weight functions. On the basis of the existing Laplacian pyramid fusion algorithm, the improved weight function can capture the more accurate image details, thereby making the fused image more detailed. In 20 sets of multi-exposure image sequences, six multi-exposure image fusion methods are compared in both subjective and objective aspects. Both qualitative and quantitative performance analysis of experimental results confirm that the proposed multi-scale decomposition image fusion method can produce high-quality HDR images.

## 1. Introduction

Due to the limited dynamic range of imaging equipment, it is impossible for existing imaging equipment to capture all the details in one scene with a single exposure. Therefore, underexposure or overexposure often occurs in daily shooting, which seriously affects the visualization of images and the display of key information. High-dynamic-range (HDR) imaging techniques overcome this limitation, but most of currently used standard monitors use low dynamic range (LDR) (Ma et al., 2015a). So, a tone mapping process is required to compress the dynamic range of HDR images for display after acquiring HDR images. Multi-exposure image fusion (MEF) methods use a cost-effective way to solve the dynamic range mismatch between HDR imaging and LDR display. Source image sequences with different exposure levels are taken as input and the brightness information in accordance with the human visual system (Ma et al., 2017) is fused with them to generate HDR images with rich information and sensitive perception.

In recent years, many MEF algorithms have been developed. Like multi-source image fusion (Jin et al., 2021a), MEF algorithms are usually divided into four categories (Liu et al., 2020): spatial domain methods, transform domain methods, the combination of spatial domain and transform domain methods and deep learning methods (Jin et al., 2021b). This article mainly studies the MEF method in spatial domain,these methods mainly focus on providing the weighted sum of the input exposures image to obtain the fused image. Different MEF methods use different techniques to obtain the suitable weight map. Li et al. (2013) obtained the corresponding base and detail layers by decomposing the source image in two scales, and then processed them separately to obtain the final fusion image. Liu and Wang (2015) applied dense scale invariant feature transform (SIFT) (Liu et al., 2016) to obtain both contrast and spatial consistency weights based on local gradient information. Mertens et al. (2010) applied multi-resolution exposure sequences to Laplacian pyramid-based image fusion. The weighted average value was first calculated from the weighted values determined by contrast, saturation and good exposure, and then applied to obtain the pyramid coefficients. Finally, image fusion was achieved by reconstructing the obtained pyramid coefficients. Shen et al. (2014) proposed an exposure fusion method based on hybrid exposure weights and an improved Laplacian pyramid. This method considers the gradient vectors between different exposure source images, and uses an improved Laplacian pyramid to decompose input signals into both base and detail layers. Shen et al. (2011) proposed a probability model of MEF. According to the two quality indicators of both local contrast and color consistency of source image sequences, the generalized random walk framework was first used to calculate the optimal probability set. Then, the obtained probability set was used as the corresponding weights to realize image Fusion. Fei et al. (2017) applied an image smoothing algorithm based on weighted least squares to MEF for achieving detail extraction of HDR scenes. The extracted detail information was used in the multi-scale exposure fusion algorithm to achieve image fusion. So, fused images with rich colors and detailed information can be obtained. Li and Kang (2012) proposed a fusion method based on weighted sum. Firstly, three image features composed of local contrast, brightness and color differences are measured to estimate the weight, and then the weight map is optimized by recursive filter. Zhang and Cham (2012) proposed a simple and effective method, which uses gradient information to complete multi exposure image synthesis in static and dynamic scenes. Given multiple images with different exposures, the proposed method can seamlessly synthesize them under the guidance of gradient based quality evaluation, so as to produce a pleasant tone mapped high dynamic range image. Ma et al. (2017) proposed a method based on image structure block decomposition, which represents the image block with average intensity, signal intensity and signal structure, and then uses the intensity and exposure factor of the image block for weighted fusion, which can be used for both static scene fusion and dynamic scene fusion. Moriyama et al. (2019) proposed to use the light conversion method of preserving hue and saturation to generate a new multi exposure image for fusion, realize brightness conversion based on local color correction, and obtain the fused image by weighted average (weight is calculated by saturation). Wang and Zhao (2020) proposed using the super-pixel segmentation method to divide the input image into non overlapping image blocks composed of pixels with similar visual attributes, decompose the image block into three independent components: signal intensity, image structure and intensity, and then fuse the three components respectively according to the characteristics of human visual system and the exposure level of the input image. Qi et al. (2020) used the exposure quality a priori to select the reference image, used the reference image to solve the ghosting problem in the dynamic scene in the structural consistency test, and then decomposed the image by using the guidance filter, and proposed a fusion method combining spatial domain scale decomposition, image block structure decomposition and moderate exposure evaluation. Li et al. (2020) proposed a multi exposure image fusion algorithm based on improved pyramid transform. The algorithm improves the local contrast information of the image by using the adaptive histogram equalization algorithm, and calculates the image fusion weight coefficient with good contrast information, image entropy and exposure. Hayat and Imran (2019) proposed a ghosting free multi exposure image fusion technology based on dense sift descriptor and guided filter. Ulucan et al. (2020) proposed a new, simple and effective still image exposure fusion method. This technique uses weight map extraction based on linear embedding and watershed masking. Xu et al. (2021). Proposed a new multi exposure image fusion method based on tensor product and tensor singular value decomposition. A new fusion strategy is designed by using tensor product and t-svd. The luminance and chrominance channels are fused respectively to maintain color consistency. Finally, the chrominance and luminance channels are fused to obtain the fused image.

Both multi-scale decomposition method and fusion strategy of multi-scale coefficients determine the performance of the image fusion framework based on multi-scale decomposition. Pyramid transformation is a commonly used multi-scale decomposition method. Due to different scales and resolutions, the corresponding decomposition layer has different image feature information. In addition, the weight function design of feature extraction plays a decisive role in the final fusion result. Therefore, this article, proposes a fast and effective image fusion method based on improved weight function. The fusion weight map is calculated through the evaluation of exposure moderation and relative brightness. Combined with pyramid multi-scale decomposition, images with different resolutions are fused to generate the required high dynamic range image.

The rest of this article is organized as follows. The second section describes the overall process of the fusion algorithm; The third section is a detailed explanation of the weight function; The fourth section describes the process of image Gaussian pyramid decomposition and Laplace pyramid decomposition; The fifth section is the experimental results and analysis; The sixth section is the summary of this article.

## 2. Workflow of Image Fusion Algorithm

**Figure 1:**
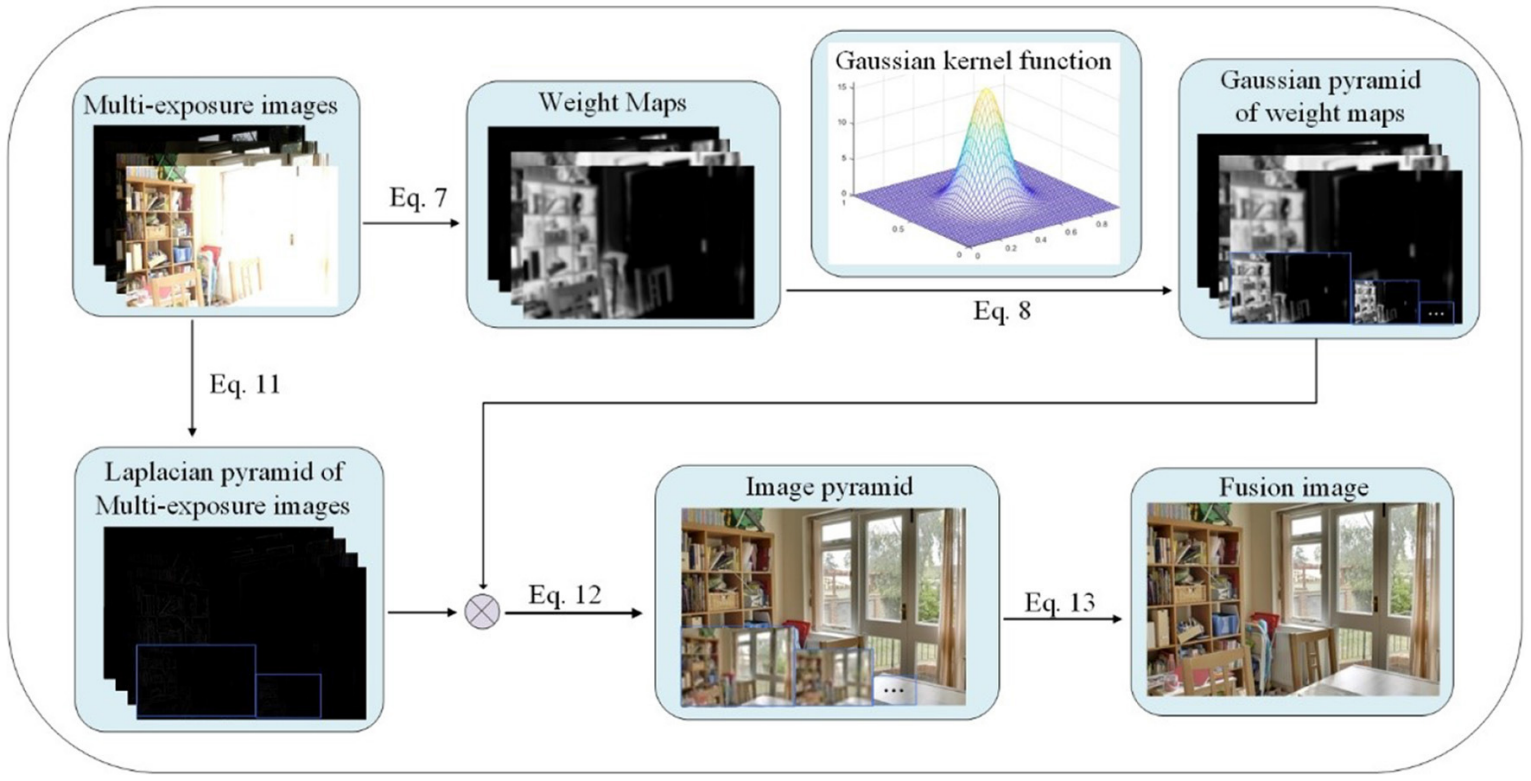
The workflow of the proposed image fusion based on improved weight function.

MEF aims to generate an image containing the best pixel information from a series of images with different exposure levels. The pixel-based MEF performs weighted image fusion as follows.


(1) $$\begin{array}{l} FI\left(x,y\right)=\overset{N}{\underset{n=1}{\sum }}W_{n}\left(x,y\right)I_{n}\left(x,y\right)\; \end{array}$$

where *FI* represents the fusion image, (*x, y*) represents pixel coordinates, *N* represents the number of images, *I*_*n*_ represents the pixel intensity of the *n*th image, and *W*_*n*_ represents the pixel weight of the *n*th image. The workflow of the proposed image fusion based on improved weight function is shown in Figure 1. Equation (7), Equation (8) and other symbols in Figure 1 correspond to the formula below, indicating that the operation corresponding to the equation has been performed. The symbol before Equation (12) in Figure 1 represents the multiplication sign.

## 3. Weight Function

As the core part of the proposed image fusion method, a reasonable weight function is designed based on the appropriate evaluation of exposure levels (Shen-yu et al., 2015). Gray value, as an important measure of image visible information, usually determines the fusion weight based on the distance between image gray and 0.5, but this single index will cause the loss of information of the fused image and some areas of the image are dark. Using the Evaluation of Moderate Exposure, the fusion weight is determined by the gray mean value of the multi exposure image at a certain point and the distance from 0.5 to retain more image information. Additionally, the relative brightness is applied to measure the corresponding weight.

### 3.1. Evaluation of Moderate Exposure

In the evaluation process, the brightness and darkness changes of different pixels obtained by the limited sampling of a scene are analyzed, and each image pixel value in the scene under the optimal moderate exposure is estimated. The differences between the pixel values of each input image and the corresponding optimal pixel values are compared to evaluate moderate exposure. The evaluation value can be directly used as the corresponding weight value for image fusion. For N images with different exposures from the same scene, *I*_*n*_(*x, y*) represents the pixel value at the coordinate (*x, y*) of the *n*th image, and the evaluation indicator of moderate exposure is the sum of weights used to obtain the fused image.


(2) $$\begin{array}{l} W_{\text{1},n}\left(x,y\right)=\exp\left\{-\frac{{\left(I_{n}\left(x,y\right)-\mu \left(x,y\right)\right)}^{2}}{2\delta^{2}}\right\}\; \end{array}$$

(3) $$\begin{array}{l} \mu \left(x,y\right)=\left(1-\beta \right)*0.5+\beta *\bar{I}\left(x,y\right)\; \end{array}$$

(4) $$\begin{array}{l} \bar{I}\left(x,y\right)=\frac{1}{N}\overset{N}{\underset{n=1}{\sum }}I_{n}\left(x,y\right)\; \end{array}$$

In Equation (2), μ(*x, y*) represents the optimal pixel value of the pixel at the coordinate (*x, y*) of the image, which is estimated by Equation (3). On one hand, the value of μ(*x, y*) should be around 0.5, which can ensure ideal human visual experience. On the other hand, in order to reflect the real-world light-dark contrast information, it is necessary to approximate the brightness information from the limited sampling of the scene. Therefore, the average value of each pixel in the images with different exposures is calculated by Equation (4). μ(*x, y*) is the weighted sum of 0.5 and this average value. The weight factor β is a balance parameter between detail information and light-dark contrast information.

### 3.2. Relative Brightness

The evaluation indicator of moderate exposure cannot well capture the information from dark areas of long-exposure images or bright areas of short-exposure images. Therefore, the relative brightness proposed by Lee et al. (2018) is added as another weight indicator. Specifically, when the overall image is bright (long exposure), the relatively dark areas are given greater weights. Conversely, when the overall image is dark (short exposure), the relatively bright areas are given greater weights. The average pixel intensity of the *n*th image is denoted as *m*_*n*_. When *I*_*n*_(*x, y*) is close to 1 − *m*_*n*_, the corresponding weight should be relatively large. Therefore, the relative brightness can be expressed as follows.


(5) $$\begin{array}{l} W_{2,n}\left(x,y\right)=\exp\left\{-\frac{{\left(I_{n}\left(x,y\right)-\left(1-m_{n}\right)\right)}^{2}}{2{\delta_{n}}^{2}}\right\} \end{array}$$

In addition, when the adjacent exposed images and the input images have relatively large differences, the different objects in the two images are often in a good exposure state. Therefore, when the average brightness *m*_*n*_ of the *n*th image considerably differs from the average brightness *m*_*n*−1_,*m*_*n*+1_ of adjacent images, a larger δ_*n*_ value is given.α is a constant with a value of 0.75. δ_*n*_ controls the weight according to the different *m*_*n*_ values of the image, which can be expressed as follows.


(6) $$\delta_{n}=\{\begin{array}{l} 2\alpha *(m_{n+1}-m_{n}),n=1\\ \alpha *(m_{n+1}-m_{n-1}),1<n<N\\ 2\alpha *(m_{n}-m_{n-1}),n=N \end{array}$$

Therefore, the final weight function can be expressed as follows.


(7) $$\begin{array}{l} W_{n}\left(x,y\right)=W_{1,n}\left(x,y\right)*W_{2,n}\left(x,y\right) \end{array}$$

## 4. Multi-Scale Image Decomposition

Because the pixels of the image are closely related, it is more reliable to use a wider range of pixels to calculate the fusion weight. In addition, in the real world, objects have different structures at different scales. This shows that if you observe the same object from different scales, you will get different results. Therefore, in the case of multi-scale decomposition, using the image pyramid to calculate the result image will get better fusion results.

Gaussian pyramid decomposition is first performed on the weight map and the multi-exposure image sequences. Then the Laplacian pyramid decomposition is applied to the multi-exposure image sequences. After the Gaussian pyramid and Laplacian pyramid of the image are fused between the corresponding layers, the upper layer image of the fused pyramid is up-sampled, and the up-sampled image is added to the lower layer image to obtain an image with the equal size of the image to be fused.

### 4.1. Gaussian Pyramid Decomposition

The Gaussian pyramid obtains a series of down-sampled images through Gaussian smoothing and sub-sampling. Gaussian kernel is first used to convolve the image of the *l* layer, and then all even rows and columns are deleted to obtain the image of the *l* + 1 pyramid layer. The decomposition algorithm is shown as follows.


(8) $$\begin{array}{l} \begin{array}{c} G_{l}\left(x,y\right)=\overset{2}{\underset{m=-2}{\sum }}\overset{2}{\underset{n=-2}{\sum }}w\left(m,n\right)G_{l-1}\left(2x+m,2y+n\right)\\ \left(0\leq l\leq L_{ev}-1,0\leq x\leq C_{l}-1,0\leq y\leq R_{l}-1\right) \end{array} \end{array}$$

where *G*_*l*_ is the image of the *l*th layer of the Gaussian Pyramid, *C*_*l*_, *R*_*l*_ is the total number of rows and columns of the *l*th layer image, *w*(*m, n*) is the value of the *m*th row and *n*th column of the Gaussian filter template, *L*_*ev*_ represents the number of Gaussian pyramid layers, and the maximum decomposable number of layers is log_2_[min(*C*_0_, *R*_0_)].

### 4.2. Laplace Pyramid Decomposition

The Gaussian pyramid obtained by Gaussian convolution and downsampling often loses detailed image information. Therefore, Mertens et al. (2010) introduced Laplacian pyramid to restore detailed image information. The image of each layer of Gaussian pyramid subtracts the predicted image obtained after the upsampling and Gaussian convolution of the upper layer image to obtain a series of difference images, which are the Laplacian decomposition images. First, the upsampling process is expressed as follows


(9) $$\begin{array}{l} expand\left({\hat{G}}_{l}\left(x,y\right)\right)=4\overset{2}{\underset{m=-2}{\sum }}\overset{2}{\underset{n=-2}{\sum }}{\hat{G}}_{l}\left(\frac{\left(x+m\right)}{2},\frac{\left(y+n\right)}{2}\right)w\left(m,n\right) \end{array}$$

(10) $${\hat{G}}_{l}(\frac{\left(x+m\right)}{2},\frac{\left(y+n\right)}{2})=\{\begin{array}{l} G_{l}(\frac{\left(x+m\right)}{2},\frac{\left(y+n\right)}{2}),\frac{\left(x+m\right)}{2},\frac{\left(y+n\right)}{2})\in z\\ 0,else \end{array}$$

where *Z* represents an integer, $expand\left({\hat{G}}_{l}\left(x,y\right)\right)$ indicates that an upsampling operation is performed on the *l*th layer of Gaussian pyramid. As shown in Equation (11), the image *G*_*l*_ of the *l*th layer of Gaussian pyramid subtracts $expand\left({\hat{G}}_{l}\left(x,y\right)\right)$ to obtain the *l*th layer image *L*_*l*_ containing detailed information.


(11) $$L_{l}=\{\begin{array}{l} G_{l}-expand({\hat{G}}_{l}(x,y)),0\leq l\leq L_{ev}-1\\ G_{L_{ev}},l=L_{ev} \end{array}$$

**Figure 2:**
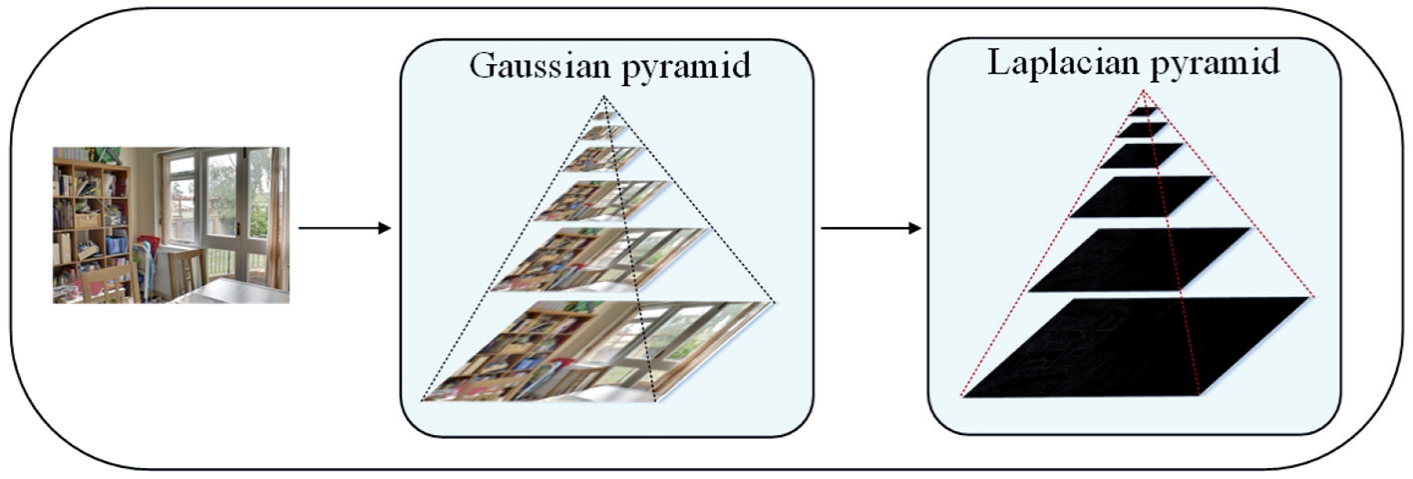
The Laplace decomposition process of the image.

The Laplace decomposition process of the image is shown in Figure 2. In this article, the number of layers of image pyramid is 7.

### 4.3. Image Fusion and Reconstruction

According to the above process, the Gaussian pyramid of the weighted image and the Laplacian pyramid of multi-exposure image sequences are first obtained, and then fused between the corresponding layers.


(12) $$\begin{array}{l} FI_{l}=\overset{N}{\underset{k=1}{\sum }}W_{k,l}L_{k,l},0\leq l\leq L_{ev}-1 \end{array}$$

*FI*_*l*_ represents the fused image data of the *l*th layer. *W*_*k,l*_ represents the *l*th layer data of the *k*th weighted image. *L*_*k,l*_ represents the *l*th layer data of the Laplacian pyramid of the *k*th multi-exposure image. *L*_*ev*_ represents the total number of pyramid layers. *N* represents the number of images. The upper layer image of the fused pyramid is first upsampled, and then expanded and added to the lower layer image to obtain an image with the equal size of the image to be fused as follows.


(13) $$\begin{array}{l} H=\overset{0}{\underset{l=L_{ev}-2}{\sum }}FI_{l}+up\left(FI_{l+1}\right) \end{array}$$

where *FI*_*l*_ represents the *l*th layer image of the fused pyramid, *up* represents upsampling, *L*_*ev*_ represents the number of pyramid levels, and *H* represents the final fusion image. The overall workflow of the proposed method is shown in Algorithm 1.

**Algorithm 1:**
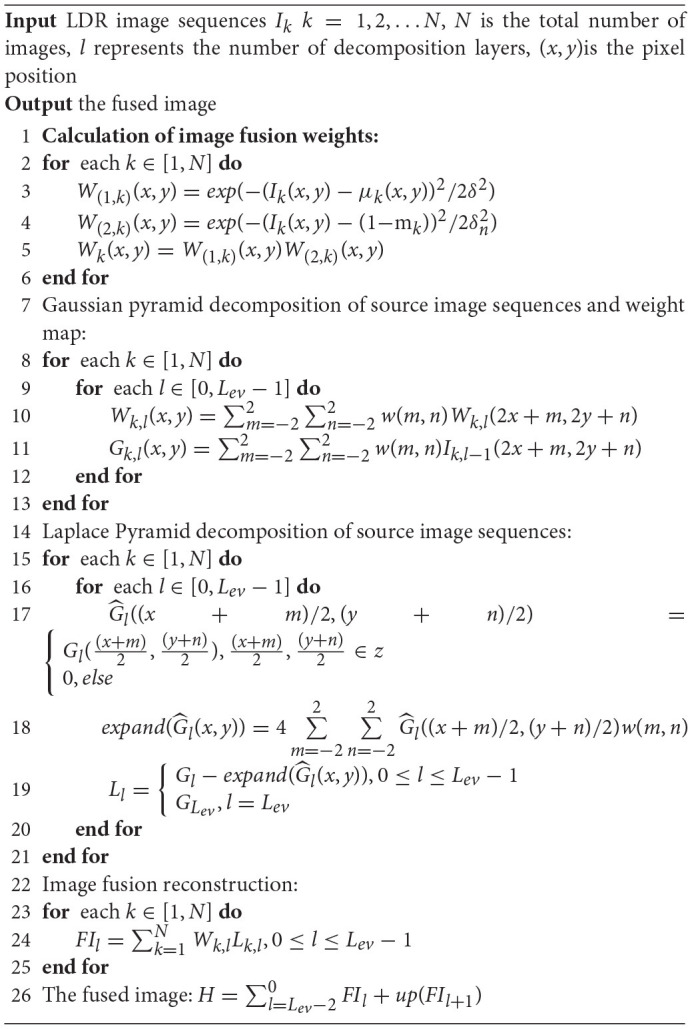
Multi-exposure image fusion algorithm based on improved weight function.

## 5. Experimental Results and Analysis

20 sets of multi-exposure image sequences, involving *Arno, Balloons, Cave, ChineseGarden*, etc., are applied to comparative experiments. The proposed method is subjectively and objectively compared with six existing MEF methods, including MESPD (Li et al., 2021), GD-MEF (Zhang and Cham, 2012), Fmmr (Li and Kang, 2012), DSIFT (Liu and Wang, 2015), GFF (Li et al., 2013), SPD-MEF (Ma et al., 2017) and PMEF (Qi et al., 2020). All experiments were performed in the matlab2019 environment on an Intel I7 9750H@2.60Ghz laptop with 8.00GB RAM. The relevant parameters are set to δ = 0.2, β = 0.5 and α = 0.75.

### 5.1. Subjective Comparison

**Figure 3:**
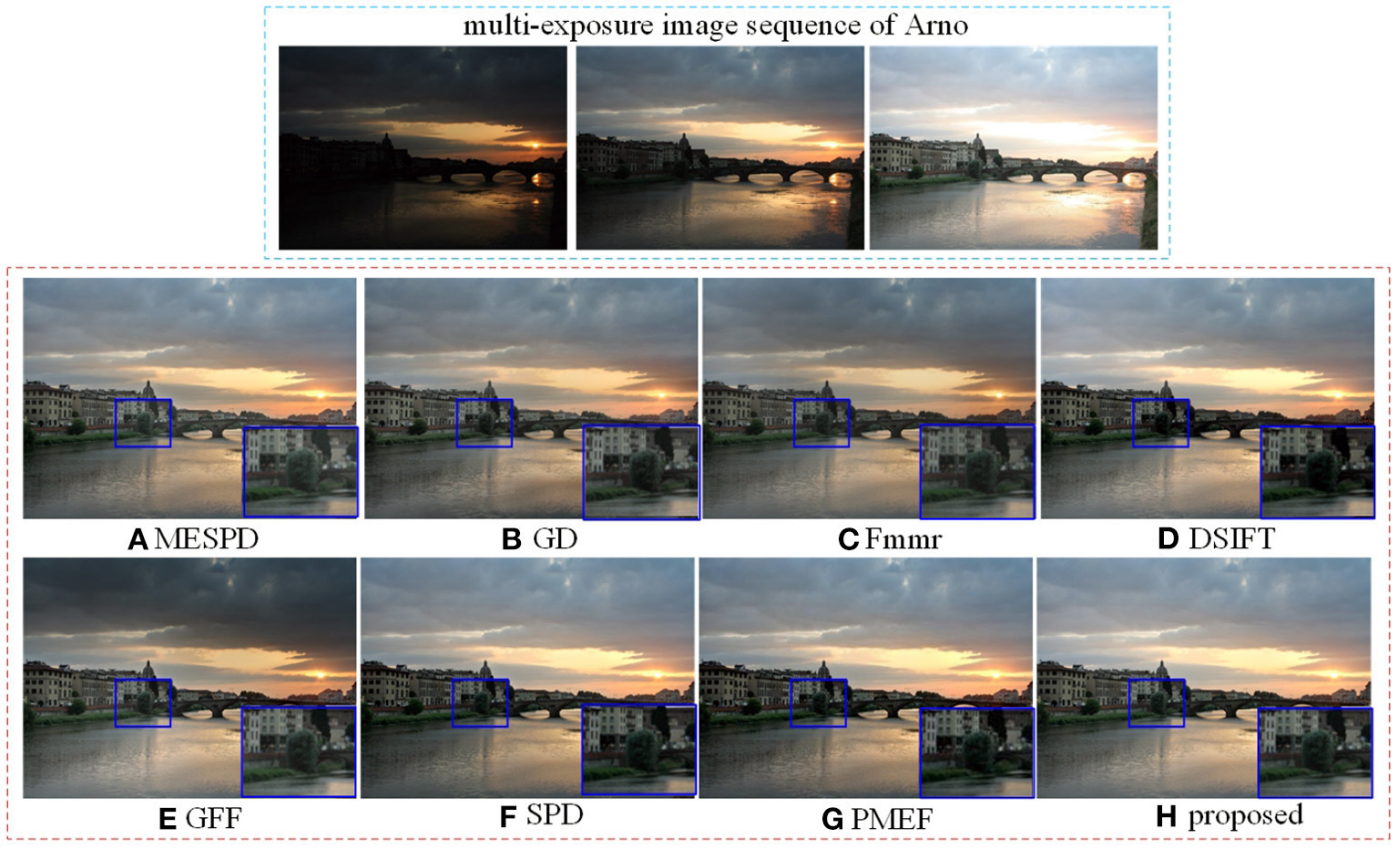
Comparison of Arno scene experiment results of different methods.

Firstly, experiments are carried out on the “*Arno*” scene, and the fusion results of different algorithms are shown in Figure 3. It is not difficult to see that when dsift processes the clouds in the right sky, it is generally dark and can not capture the details of the clouds well. The GFF and SPD algorithms, when dealing with the bridge, have the problems of low brightness, resulting in the loss of detail information and poor visual effect. GD, PMEF and the algorithm proposed in this article can maintain the uniformity of the overall brightness of the image while retaining more details, and the visual effect is excellent.

**Figure 4:**
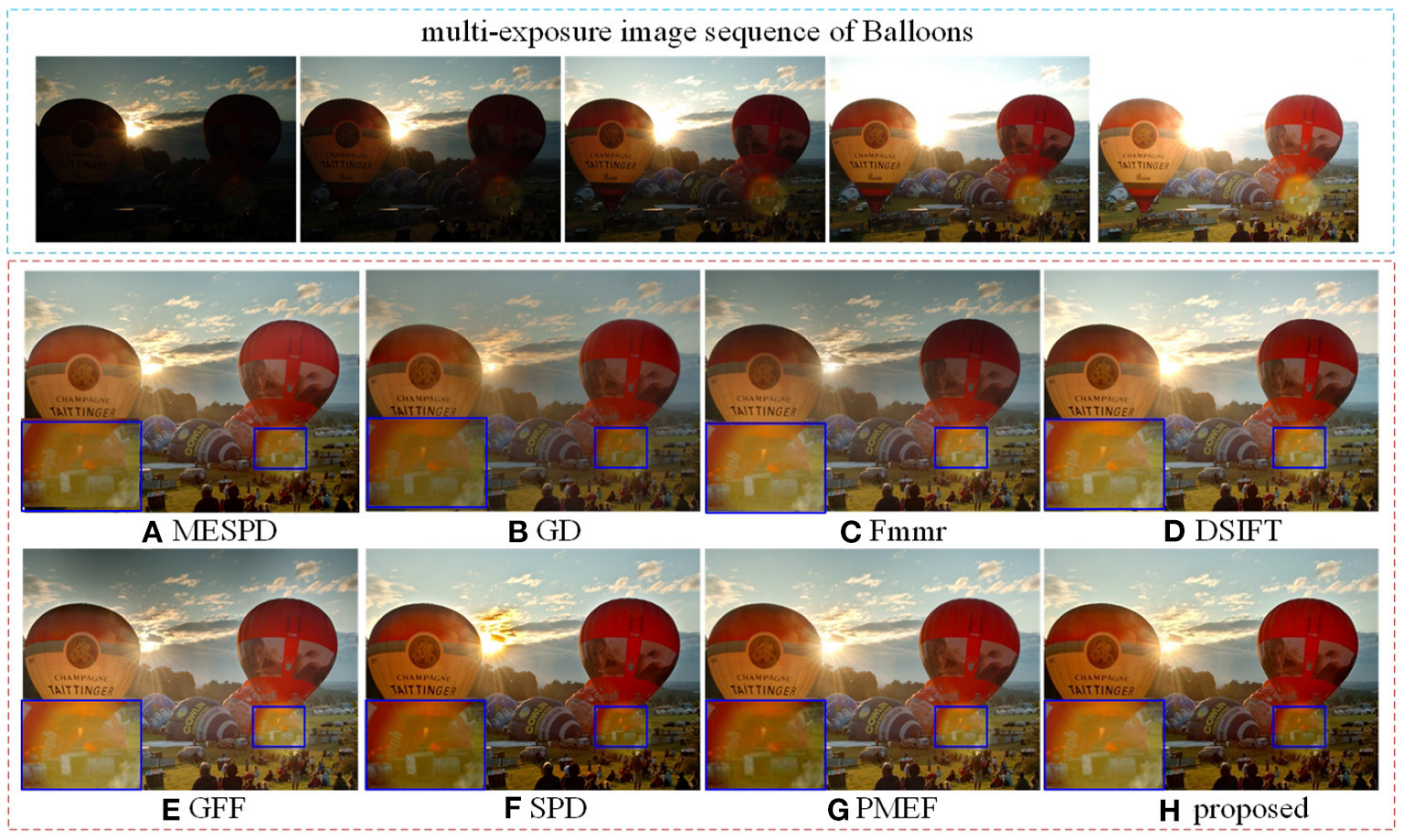
Comparison of Ballons scene experiment results of different methods.

The experimental results of “*Balloons*” scene are shown in Figure 4. The fusion results of GD, Fmmr and PMEF are dark. The details of clouds at the sunset are well captured, which means the details of the overexposed image areas can be well captured. But the overall scene is dark, resulting in the detail loss of underexposed image areas. The image fused by GFF has a slight halo on the edge of the hot air balloon. Additionally, part of the sky is dark and the image color is slightly distorted. The sunset area of the image fused by SPD is abnormal. In addition, the image color is seriously distorted, which seriously affects the overall performance of the fused image. When comparing the enlarged details, MESPD, GD, Fmmr, SPD, and PMEF have low brightness, poor visibility and serious loss of details in this area.

**Figure 5:**
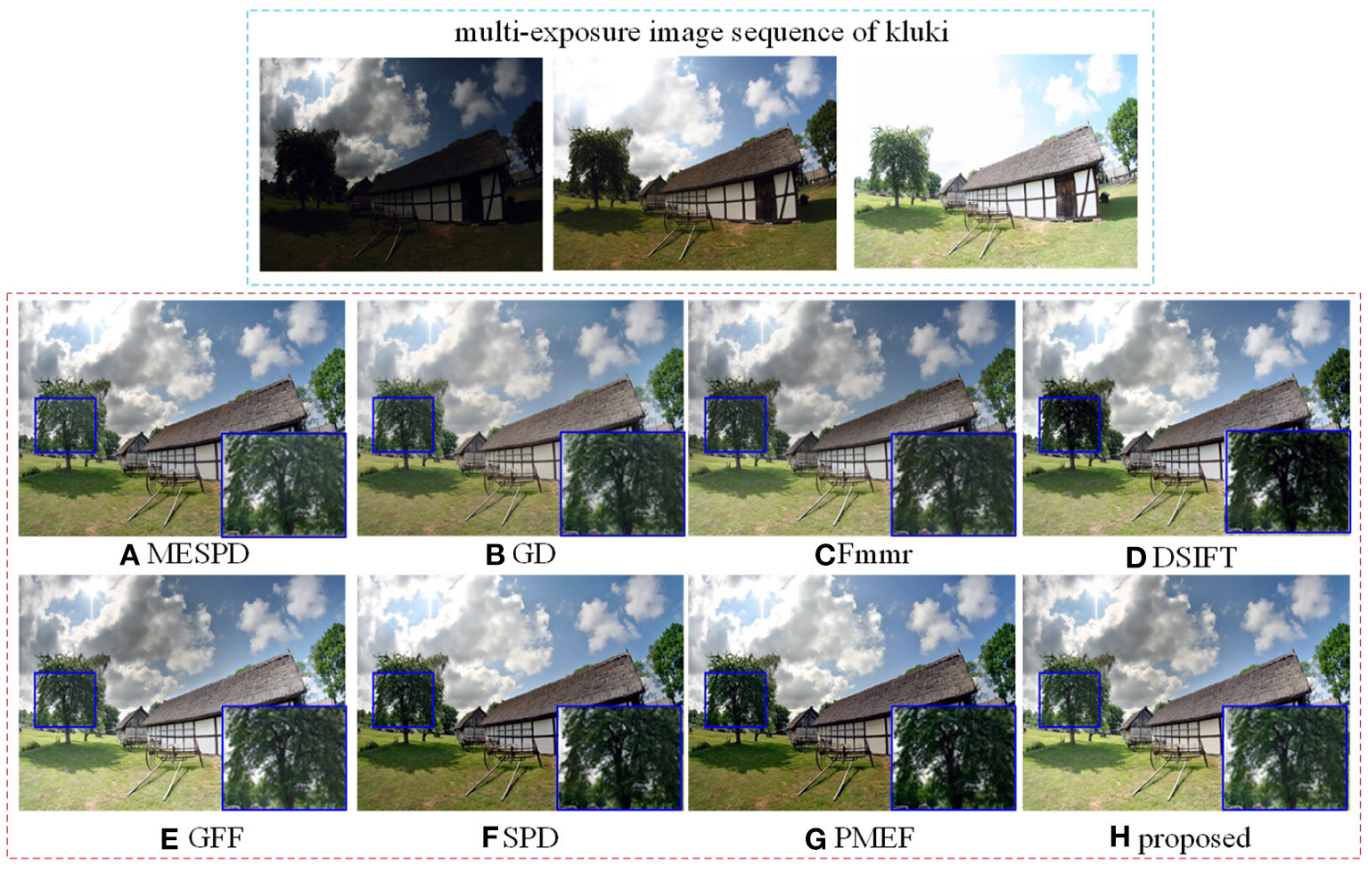
Comparison of kluki scene experiment results of different methods.

In the experimental results of the “*Kluki*” scene, as shown in Figure 5, the saturation of SPD and PMEF is too high, resulting in some distortion of the color of the resulting image, and poor retention of the details of the clouds in the sky; Other algorithms retain the details of the clouds, and the visual effect is good. In contrast, the fusion results obtained by the proposed method and DSIFT consider the details of the bright and dark areas of the scene. So, the corresponding colors are real, the contrast is clear, and the visual performance of the fused images is good. From the enlarged details of the trees on the left, dsift, SPD, and PMEF have the problems of low brightness and high saturation, resulting in poor retention effect of details.

### 5.2. Objective Evaluation Indicator Analysis

**Figure 6:**
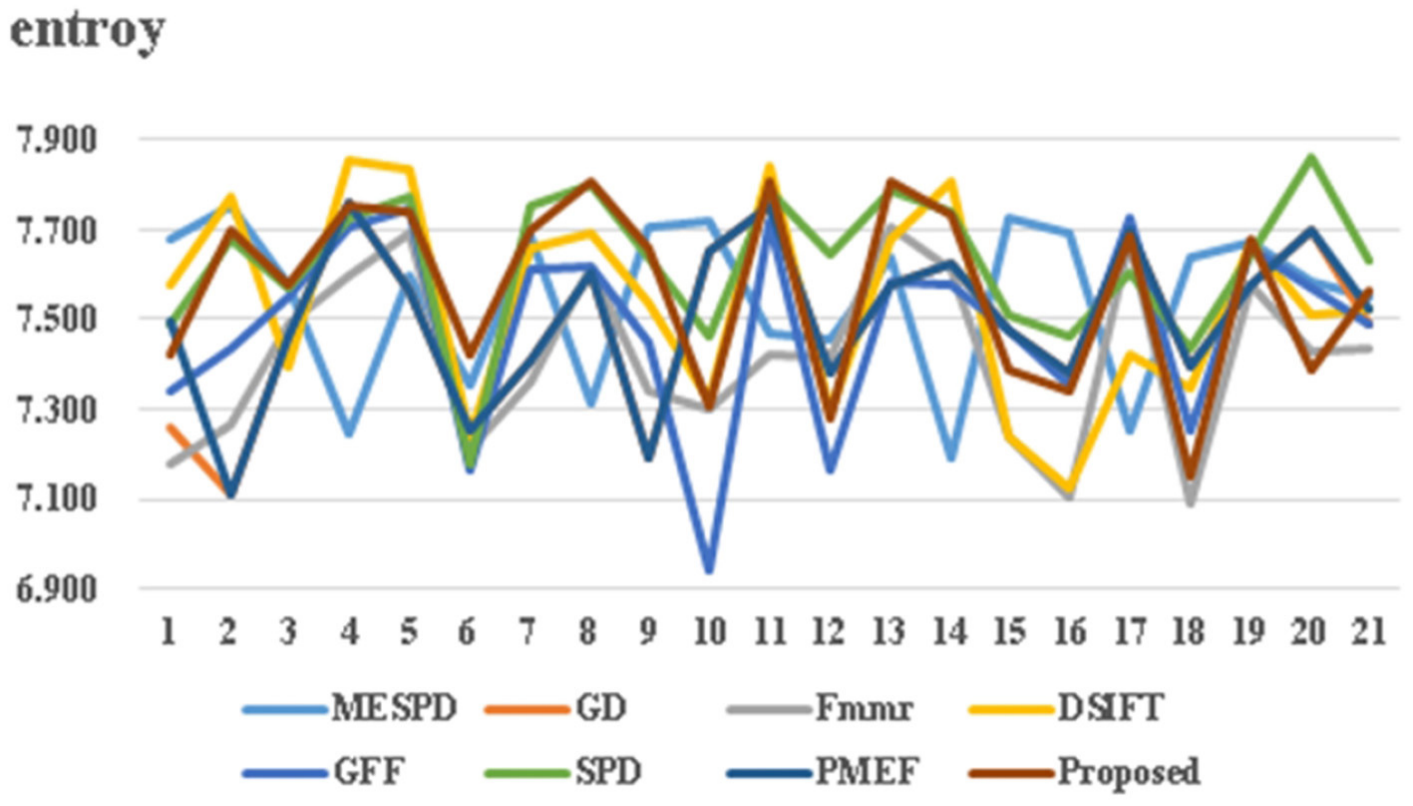
Information entropy comparison of seven fusion methods.

**Figure 7:**
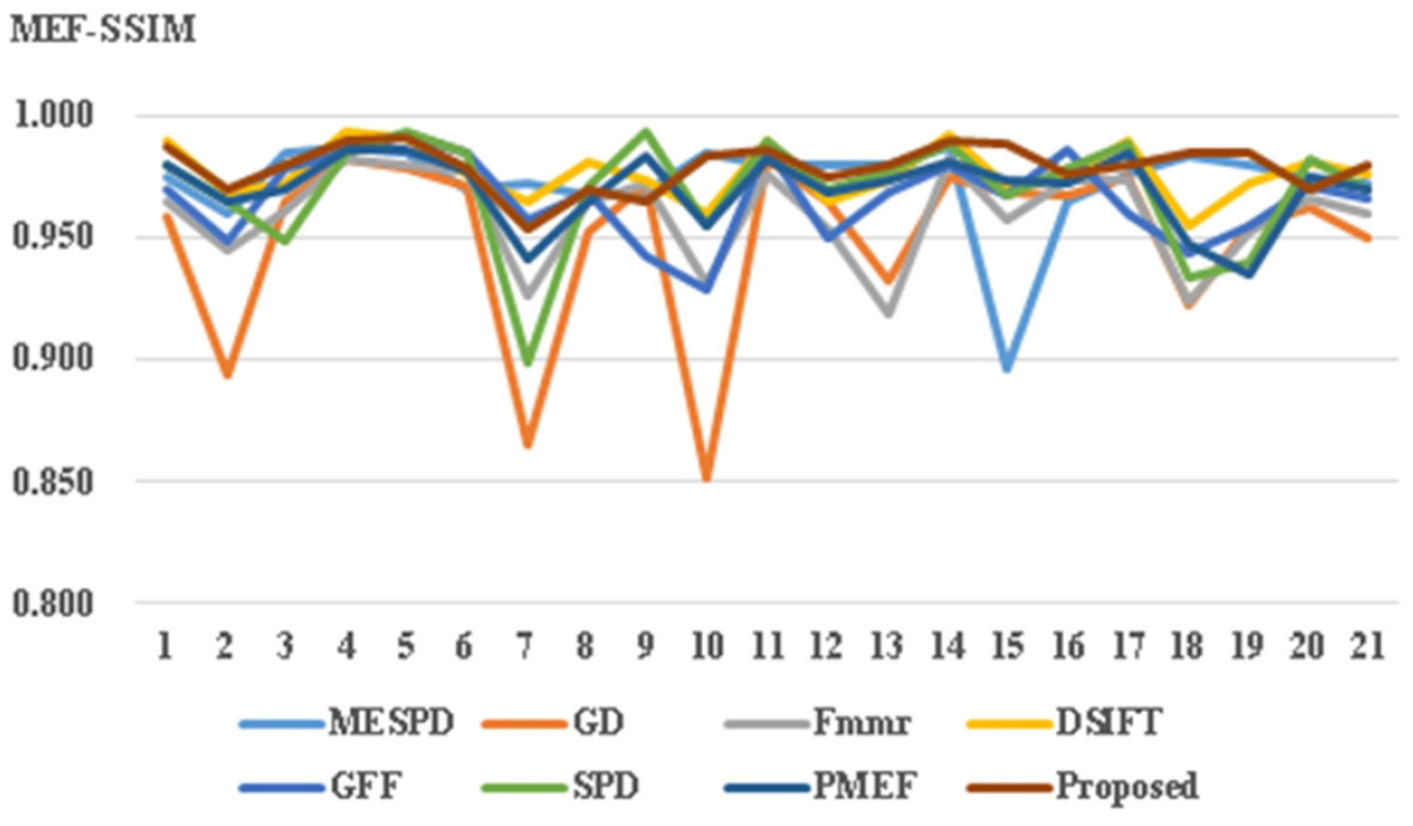
Comparison of MEF-SSIM indexes of seven fusion methods.

This article uses both structural similarity index (SSIM) and image information entropy for objective evaluation. As shown in Figure 6 and Tables 1, 2, the results confirm that the propose method achieves good performance in both subjective and objective evaluations. The abscissa in Figure 6 represents the value of information entropy, and the abscissa in Figure 7 represents the value of structural similarity; In addition, the ordinates of the two figures are the same: 1-20 represents different multi exposure sequences, and 21 represents the average value.

**Table 1:** Information entropy comparison of seven fusion methods.

**Methods**	**MESPD**	**GD**	**Fmmr**	**DSIFT**	**GFF**	**SPD**	**PMEF**	**Proposed**
1. Arno	**7.679**	7.258	7.175	7.581	7.343	7.490	7.498	7.424
2. Balloons	7.752	7.113	7.264	**7.773**	7.435	7.676	7.113	7.703
3. Cave	7.577	7.463	7.488	7.396	7.551	7.572	7.463	**7.579**
4. ChineseGarden	7.248	7.762	7.598	**7.852**	7.704	7.728	7.762	7.752
5. Church	7.601	7.565	7.693	**7.838**	7.744	7.774	7.565	7.737
6. Farmhouse	7.356	7.251	7.214	7.237	7.162	7.176	7.251	**7.424**
7. House	7.687	7.408	7.360	7.659	7.609	**7.755**	7.408	7.697
8. Kluki	7.312	7.603	7.620	7.696	7.618	7.801	7.603	**7.809**
9. Lamp	**7.705**	7.195	7.343	7.535	7.452	7.642	7.195	7.657
10. Landscape	**7.720**	7.655	7.303	7.322	6.938	7.460	7.655	7.305
11. Laurenziana	7.469	7.751	7.423	**7.840**	7.717	7.786	7.751	7.805
12. Lighthouse	7.458	7.384	7.413	7.292	7.167	**7.645**	7.384	7.283
13. MadisonCapitol	7.637	7.576	7.705	7.678	7.586	7.787	7.576	**7.809**
14. Mask	7.190	7.623	7.610	**7.811**	7.580	7.738	7.623	7.735
15. Office	**7.728**	7.473	7.236	7.236	7.473	7.507	7.473	7.387
16. Ostrow	**7.694**	7.382	7.105	7.122	7.356	7.460	7.382	7.342
17. Room	7.254	7.701	7.681	7.424	**7.729**	7.608	7.701	7.687
18. Set	**7.639**	7.394	7.092	7.347	7.255	7.438	7.394	7.150
19. Tower	7.672	7.576	7.579	7.675	7.657	7.646	7.576	**7.676**
20. Venice	7.584	7.701	7.430	7.513	7.571	**7.861**	7.701	7.387
21. Average	7.548	7.492	7.438	7.526	7.493	**7.633**	7.526	7.567

*The bold value indicates the highest objective evaluation index value in this group of experiments*.

**Table 2:** Comparison of MEF-SSIM indexes of seven fusion methods.

**Methods**	**MESPD**	**GD**	**fmmr**	**DSIFT**	**GFF**	**SPD**	**PMEF**	**Proposed**
1. Arno	0.975	0.958	0.965	**0.989**	0.969	0.980	0.98	0.987
2. Balloons	0.959	0.893	0.945	0.968	0.948	0.965	0.965	**0.970**
3. Cave	**0.984**	0.964	0.961	0.972	0.978	0.948	0.969	0.980
4. ChineseGarden	0.987	0.982	0.982	**0.993**	0.984	0.985	0.986	0.989
5. Church	0.985	0.978	0.979	0.991	0.992	**0.993**	0.986	0.991
6. Farmhouse	0.970	0.971	0.977	0.976	0.985	**0.984**	0.977	0.978
7. House	**0.972**	0.865	0.926	0.964	0.957	0.898	0.941	0.953
8. Kluki	0.967	0.952	0.965	**0.981**	0.968	0.971	0.965	0.970
9. Lamp	0.968	0.972	0.972	0.973	0.942	**0.993**	0.983	0.965
10. Landscape	0.984	0.851	0.931	0.960	0.929	0.954	0.955	**0.983**
11. Laurenziana	0.98	0.982	0.976	0.989	0.987	**0.990**	0.982	0.986
12. Lighthouse	**0.979**	0.964	0.953	0.965	0.950	0.970	0.968	0.975
13. MadisonCapitol	**0.980**	0.932	0.918	0.973	0.968	0.977	0.973	**0.980**
14. Mask	0.987	0.975	0.982	**0.992**	0.979	0.988	0.981	0.990
15. Office	0.896	0.968	0.957	0.971	0.967	0.967	0.973	**0.988**
16. Ostrow	0.965	0.967	0.973	0.974	**0.986**	0.978	0.972	0.976
17. Room	0.976	0.975	0.973	**0.990**	0.960	0.988	0.984	0.980
18. Set	0.983	0.922	0.924	0.954	0.943	0.934	0.947	**0.984**
19. Tower	0.980	0.954	0.952	0.972	0.954	0.940	0.935	**0.985**
20. Venice	0.975	0.962	0.966	0.981	0.971	**0.982**	0.975	0.969
21. Average	0.973	0.949	0.959	0.976	0.966	0.969	0.970	**0.979**

*The bold value indicates the highest objective evaluation index value in this group of experiments*.

1) Image information entropy indicator comparison

Image information entropy is one of the important factors that determine the final effect of image fusion. The larger the information entropy, the more detailed information contained in the experimental result graph; On the contrary, the smaller the information entropy, the less detailed information contained in the experimental result graph. The evaluation results are shown in Figure 6 and Table 1. The multi exposure fusion algorithm under multi-scale decomposition is slightly lower than the SPD algorithm based on image block decomposition and better than other algorithms. This is because the SPD algorithm based on image block decomposition avoids the partial loss of information caused by up and down sampling in multi-scale decomposition, and its entropy is better than the multi exposure fusion algorithm under multi-scale decomposition. The calculation formula of image entropy is as follows.


(14) $$\begin{array}{l} H=\overset{255}{\underset{i=0}{\sum }}P_{i}\log p_{i} \end{array}$$

*P*_*i*_ represents the proportion of pixels with gray value *i* in the image.

2) MEF-SSIM comparison

This article uses the MEF quality evaluation model (Ma et al., 2015b) for evaluation. The proposed method is objectively compared with six existing MEF method. Natural images usually contain object information of different scales. Multi-scale can ensure the correlation between the pixels of different scales and optimize image fusion. Structural similarity as an index is used to measure the similarity of two images. As shown in Figure 7 and Table 2, the MEF method under multi-scale decomposition achieves the best SSIM.

From the perspective of image composition, structural information is defined as an attribute that reflects the structure of objects in the scene independent of brightness and contrast. Additionally, model distortion is treated as a combination of three different factors, brightness, contrast, and structure. The mean is used as an estimate of brightness. The standard deviation is used as an estimate of contrast. The covariance is used as a measure of structural similarity. All the definitions are shown as follows.


(15) $$\begin{array}{l} SSIM\left(x,y\right)={\left[L\left(x,y\right)\right]}^{\alpha }\cdot {\left[C\left(x,y\right)\right]}^{\beta }\cdot {\left[S\left(x,y\right)\right]}^{\gamma } \end{array}$$

(16) $$\begin{array}{l} L\left(x,y\right)=\frac{2\mu_{x}\mu_{y}+c1}{\mu_{x}^{2}+\mu_{y}^{2}+c1} \end{array}$$

(17) $$\begin{array}{l} C\left(x,y\right)=\frac{2\delta_{x}\delta_{y}+c2}{\delta_{x}^{2}+\delta_{y}^{2}+c2} \end{array}$$

(18) $$\begin{array}{l} S\left(x,y\right)=\frac{\delta_{xy}+c3}{\delta_{x}\delta_{y}+c3} \end{array}$$

*L*(*x, y*), *C*(*x, y*), and *S*(*x, y*) are the comparison results of image brightness, contrast, and structure, respectively. μ_*x*_ and μ_*y*_ are the mean values of image pixels. δ_*x*_ and δ_*y*_ are the standard deviations of image pixel values. δ_*x,y*_ is the covariance of x and y. *c*1, *c*2, and *c*3 are constants to avoid system errors when the denominator is 0. α, β, γ used to adjust the weight of each component, usually α = β = γ = 1. The structural similarity index is used for different scales, and the final image quality score is obtained through Formula (19).


(19) $$MEF-SSIM=\sum_{l=1}^{L}[SSIM_{l}{]}^{\beta_{l}}$$

where *L* is the total number of scales and β_*l*_ is the weight assigned to the *l*th scale.

### 5.3. Ablation Experiment of Weight Function

In order to prove that the weight function of two different feature indexes, moderate exposure evaluation and relative brightness, can make the multi exposure image fusion get better results. The following ablation experiments were carried out in this article. As shown in Table 3, the objective evaluation index of the fused image obtained by the improved weight function in this article performs well.

**Table 3:** Ablation experiment of weight function.

**Weight function**	**Evaluation of moderate**	**Exposure relative brightness**	**Proposed**
Entroy	7.513	7.525	**7.567**
MEF-SSIM	0.966	0.970	**0.979**

*The bold value indicates the highest objective evaluation index value in this group of experiments*.

### 5.4. Comparison and Analysis of Computational Efficiency

As shown in Table 4, The computational efficiency of the multi exposure fusion algorithm based on the improved weight function is better than the comparison algorithm. In the multi-exposure fusion algorithm based on the improved weight function, although the Laplace image pyramid is used, in the continuous down sampling, the amount of calculation increases only a little due to the doubling of the number of pixels. In addition, because the weight calculation method of this algorithm is simple and easy to calculate, it does not need additional time. Therefore, the computational efficiency of this algorithm is obviously better than other comparison algorithms.

**Table 4:** Comparison of image fusion efficiency of seven fusion methods.

**Methods**	**MESPD**	**GD**	**fmmr**	**DSIFT**	**GFF**	**SPD**	**PMEF**	**Proposed**
Average time(s)	4.974	0.983	1.090	1.569	0.691	1.361	2.814	**0.327**

*The bold value indicates the highest objective evaluation index value in this group of experiments*.

## 6. Conclusion

In this article, the weight function is improved, and the weight map is calculated by using the evaluation of moderate exposure and relative brightness. Pyramid-based multi-scale decomposition is used to fuse images with different resolutions to generate the final HDR image. The proposed method can effectively capture the rich image details and solve the issues such as splicing traces and border discontinuities in the fused image, avoiding the generation of artifacts. Both MEF-SSIM and image information entropy are used to evaluate the performance of image fusion. Experimental results confirm that the proposed method achieves good image fusion performance.

## Data Availability Statement

The raw data supporting the conclusions of this article will be made available by the authors, without undue reservation.

## Author Contributions

KX: conceptualization, funding acquisition, and supervision. QW: investigation and methodology. HX and KL: software. All authors have read and agreed to the published version of the manuscript. All authors contributed to the article and approved the submitted version.

## Conflict of Interest

The authors declare that the research was conducted in the absence of any commercial or financial relationships that could be construed as a potential conflict of interest.

## Publisher's Note

All claims expressed in this article are solely those of the authors and do not necessarily represent those of their affiliated organizations, or those of the publisher, the editors and the reviewers. Any product that may be evaluated in this article, or claim that may be made by its manufacturer, is not guaranteed or endorsed by the publisher.
